# Control and benchmarking of a 7-DOF robotic arm using Gazebo and ROS

**DOI:** 10.7717/peerj-cs.383

**Published:** 2021-03-23

**Authors:** Bowei Zhang, Pengcheng Liu

**Affiliations:** Department of Computer Science, University of York, York, UK

**Keywords:** Robotic arm, Control, Benchmarking, Accuracy, Efficiency, Robustness, Gazebo, ROS

## Abstract

The robot controller plays an important role in controlling the robot. The controller mainly aims to eliminate or suppress the influence of uncertain factors on the control robot. Furthermore, there are many types of controllers, and different types of controllers have different features. To explore the differences between controllers of the same category, this article studies some controllers from basic controllers and advanced controllers. This article conducts the benchmarking of the selected controller through pre-set tests. The test task is the most commonly used pick and place. Furthermore, to complete the robustness test, a task of external force interference is also set to observe whether the controller can control the robot arm to return to a normal state. Subsequently, the accuracy, control efficiency, jitter and robustness of the robot arm controlled by the controller are analyzed by comparing the Position and Effort data. Finally, some future works of the benchmarking and reasonable improvement methods are discussed.

## Introduction

Robot technology plays an important role in industry and life. In actual use, fully autonomous robots account for the majority (Yudha et al., 2018). Among many types of robots, the use of robotic arms is very wide. In the industry, robotic arms are used to complete tasks such as welding, assembly and labelling; In life, the robotic arm can be used for cooking, cleaning and other household service tasks (Ersen, Oztop & Sariel, 2017). However, the control of robotic arms is a challenging task both in industry and in life (Liu et al., 2020). Because the robotic arm needs to face a complex unstructured environment and various indeterminate interferences, these problems cause the sensor to obtain incomplete information or even obtain incorrect information, which makes the robotic arm unable to complete the task. Therefore, the control and use of robotic arms in unstructured environments has become a hot topic in recent years.

In general, the steps of the robot arm to complete the task can be abstracted into four steps: Operation before grabbing, grab the object, transport it after grabbing, and place it to the target location (Vernon, 2014). The first step requires the robot arm to move to the target. To achieve this, motion planning is required. Then the gripper needs to be in contact with the target object during the gripping phase. In the third step, after grasping the object, the robotic arm moves to the specified target position. The last step is to put down the object. In the whole process, it is necessary to calculate the trajectory of the robotic arm, and a controller with superior performance to complete the precise control of the robotic arm (Liu et al., 2018a; Shyam et al., 2019). Similar robotic arm simulation tasks, for example: in Militaru, Mezei & Tamas (2016) is a 7-DOF robotic arm and a commercial sensor. In order to complete the trajectory control they used MoveIt (Coleman et al., 2014) to test different planners. In Dawson et al. (2014) the researchers simulated a 5-DOF manipulator, and they exported the manipulator model to ROS for simulation. Robot operating systems (ROS) (Quigley et al., 2009) and MoveIt allow researchers to use various packages to control the robot to complete designated tasks.

Whether in simulation or actual use, achieving efficient and accurate control of the robotic arm is a challenging task (Szczçsny & Rećko, 2018). Different types of robotic arms, such as rigid robotic arms (Falco et al., 2019) or flexible robotic arms (Fang et al., 2019), have different control methods. Because the redundant robot arm with seven degrees of freedom has excellent performance in a wide range of applications, the Franka Emika Panda (Gaz et al., 2019) seven degrees of freedom robot was selected for this work. The purpose is to compare the performance of different controllers on a multi-DOF robotic arm. Compared with the non-redundant 6-DOF robotic arm, the 7-DOF redundant robotic arm has great advantages in obstacle avoidance and joint torque optimization (Zhang et al., 2019). In addition to the advantages of 7 DOF, the control mode of the multi-DOF robotic arm can provide a reference for other DOF robotic arm control. In the field of robotic arm control, except accurate control is an important requirement, the robustness of robotic arm control is also an important research topic (Cao et al., 2019). This is because uncertain factors in the real world include, but are not limited to, machine wear, gravity-affected manipulation, motor errors and noisy environments. These points are also challenging that need to be faced in the simulation of robotic arms, which means how to convert the uncertain factors in real-world environment into the parameters of the simulated environment, in order to highly fit and reflect the real-world environment.

In summary, there are many types of controllers, and there are different degrees of differences between the same types of controllers is the motivation of this article. The purpose of this article is to explore the performance differences in accuracy between the same types of basic and advanced controllers. As mentioned above, the accurate control of the robotic arm is a challenging task, and the performance of the controller can be reflected in the accuracy of controlling the robotic arm. Therefore, the accuracy test is one of the tasks in the benchmarking. Furthermore, the control efficiency is also used as a task of the benchmarking because the faster the robot arm runs, the accuracy will decrease. In the case of the same accuracy, the faster the robot arm runs, the better the performance of the controller. So, control efficiency is also used as part of the benchmark test. In addition, the jitter of the robotic arm is also one of the criteria for the performance of the controller (Banerjee, Yu & Aggarwal, 2018), so the jitter test is also part of this benchmarking. Considering that the working environment of the robot arm is usually disturbed by external factors, this became the motivation to add robustness testing to the controller benchmarking. Since the abnormal operation of the robotic arm will be involved in the test process, which may cause damage to the real robot, the simulation method is chosen to complete this work. The software used for the simulation is ROS+MoveIt+Gazebo. ROS provides a lot of toolkits and plug-ins to help complete the work, MoveIt provides motion planning functions, and Gazebo (Koenig & Howard, 2004) provides a very good simulation environment, the simulation environment can fully simulate all conditions in reality and a simple interactive interface. There are two types of controllers studied in this article: advanced controllers and basic controllers. Advanced controllers include an Active inference controller (AIC) proposed by (Pezzato, Ferrari & Corbato, 2020) and a model reference adaptive controller (MRAC), and basic controllers are joint effort controller, joint position controller and joint velocity controller provided by ROS. By completing the benchmarking of these controllers, the aim of benchmarking between the same types of controllers is achieved. AIC is an adaptive controller (Mercadé, 2018), and it can be extended to a high-DOF robotic arm. It is worth noting that AIC uses perception and biological knowledge as a basis. MRAC is a model reference adaptive controller (Zhang & Wei, 2016). Its main idea is to obtain a control signal to be applied to the robot's brake, which forces the system to operate in a manner specified by the selected reference model.

In order to achieve the purpose of benchmarking the controller, the task of benchmarking is to simulate pick and place (Nabat et al., 2005; Sugiarto & Conradt, 2017; Toan & Khoi, 2019). Four poses are designed in the experiment, three poses are used to simulate pick-and-place actions, and one initial pose is used to determine whether the controller can control the robotic arm to return to the initial state. The purpose of the design these three poses is to enable the controller to control the robot arm to run at different angles, and to prevent the controller from performing poorly in some specific positions. Then obtain the operating data (Position, Torque) of each joint of the robotic arm by using the data collection plug-in provided by ROS. Position data is used to observe whether the robot arm can reach the correct pose and the time required to reach the pose, so as to compare the control efficiency and accuracy of the controller. By analyzing Torque data, the jitter of the robotic arm can be obtained. Finally, by applying an external force to the robot arm through the built-in function of Gazebo and analyzing the time required for the robot arm to return to the normal state and the position data of the joint, the robustness of the controller can be obtained. There are two criteria for the success of the test: First, the controller can control the robot arm to reach or approach the target pose every time; second, data collection is not affected by any uncertain factors. The contribution of this article is to complete the benchmarking of the same type of controller through the designed experiment, and to analyze the test results of these controllers based on the experimental results.

The structure of the article is as follows: In the second part, we compiled the work on the development and design of the controller and some benchmarking methods. The third part describes in detail the experimental environment such as ROS, Gazebo, etc. More importantly, the specific description of the controller, as well as the inspiration and design of the experimental method. In the data analysis part, corresponding analysis and summary will be given based on the experimental results. The fourth part is the experimental result, and the fifth part is the experimental result analysis and discussion. Furthermore, some experimental deficiencies and future work will be included in this part. The last part is the conclusion of the entire article.

## Related Works

Robot controllers can be divided into two categories. One is model-based controllers, and the other is model-free controllers. Models include robot dynamics models, environment models, motor/actuator models, etc. These models will be affected by many factors in the process of establishment and use, such as machine wear and gravity (Zhang et al., 2016). In order to solve the model problem, researchers are committed to developing model-based controllers to suppress or adapt to the uncertainty of these models (Liu, Yu & Cang, 2014, 2016, 2018a, 2018b, 2018c, 2018d, 2019; Liu et al., 2018b, 2019; Huda et al., 2020). In Cao et al. (2019) an adaptive controller is proposed, which is a novel control method, which combines adaptive filtering and admittance control to overcome the shortcomings of the constant admittance controller. In another study (Sugiarto & Conradt, 2017), researchers developed a kinematic model based on general factor graphs. In order to solve the problem of fine-tuning the parameters of the kinematic model, they chose to use a data-driven learning method to complete the task of fine-tuning the parameters. Also in (Toan & Khoi, 2019), researchers used the pick and place task to test a fuzzy-based admittance controller. In Kang et al. (2019), it is also about the controller design of human-robot interaction. The researchers proposed two types of admittance control schemes, one for direct intent and the other for indirect intent. When benchmarking the controller, the researchers selected a 6-DOF robot arm and set up a series of tasks. To solve the problem of parameter uncertainty (Abraham et al., 2020), the researchers proposed a model-predictive path integral control strategy.

Another type of controller is a model-free controller. The characteristic of a model-free controller is that it usually does not consider the accuracy of the model and the uncertainty of the model, but achieves the purpose of control through learning or training (Popović et al., 2011). In recent years, the popularity of machine learning has continued to increase, and many developers have begun to use machine learning technology to develop new control systems in order to improve the performance of the controller (Williams et al., 2017; Wu et al., 2019b). Not only used in model-free control systems, but also in the application of machine learning technology in model-based control systems (Lee et al., 2020). But not all model-free controllers use machine learning technology. In Wang, Wang & Wang (2019) they designed an impedance controller based on torque feedback. For the rigid robot in Esmaeili et al. (2019), the researchers proposed an observer-based data-driven model-free adaptive terminal sliding mode controller. In addition, reinforcement learning is also applied to model-free control. In Perrusquía, Yu & Soria (2019), the researchers proposed a model-free robot interactive control method using reinforcement learning. In Wu et al. (2019a) proposed a model-free adaptive iterative learning controller based on an iterative feedback adjustment algorithm.

In summary, different types of controllers have different problems (Zhou et al., 2017; Zhang, Hu & Gow, 2020). Model-based controllers need to build models to suppress or adapt to the influence of many uncertain factors (wear, gravity, etc.) in robot applications. The design of a model-free controller needs to consider developing the controller without using a model and effectively controlling the operation of the robot arm through the controller. Although the problems to be solved by the two types of controllers are different, the fundamental purpose is to improve the accuracy of the controller to control the robotic arm.

## Methodology and Experiment Design

### ROS+Gazebo

ROS and Gazebo are the main tools of this experiment.

Gazebo is a 3D dynamic simulator that can accurately and efficiently simulate robots in complex indoor and outdoor environments. Gazebo uses a distributed architecture with independent libraries for physical simulation, user interfaces, communication and sensor generation.

### Controllers

#### Base controllers

The three base controllers used for benchmarking come from ROS: JointPositionController, JointVelocityController and JointEffortController. These controllers are included in the ROS control package:

- JointPositionController: The input data is position (radians (or) meters). The data calculation of the controller is to convert the difference between the goal position and the current position into force data through the PID controller.
- JointVelocityController: The input data is the velocity (radians/sec (or) meters/sec). The data calculation of the controller is to convert the difference between the goal velocity and the current velocity into force data through the PID controller.
- JointEffortController: The input data is the effort (force (or) torque). The controller does not need the data conversion of the PID controller, because its input data and output data are all efforts.

It is worth noting that the first two controllers will be affected by the PID controller, so in the subsequent experiments will involve the adjustment of the PID parameters, so that the controller can achieve better results.

### Advanced controllers

The two advanced controllers selected for this article are AIC and MRAC.

- AIC: the control scheme is based on Karl Friston’s free energy principle (FEP) (Friston, Mattout & Kilner, 2011). The active inference framework uses FEP to explain perceptions and actions, and the subject has a world model that is optimized using sensory input by predicting and interpreting its feelings. The main idea of active inference is that the brain can manipulate sensory input and brain state to minimize prediction errors (16). In this way, by changing the sensory input of behavior or modifying its beliefs about the state of the world, behavior and perception can be used to minimize free energy. AIC can be used as an adaptive control scheme for robotic arms, which is easily scalable to high DOF, and it maintains high performance even in the presence of large unmodeled dynamics.
- MRAC: model reference adaptive control is a direct adaptive strategy with some adjustable controller parameters and adjustment mechanisms for adjusting them (Jain & Nigam, 2013). Different from commonly used PID controllers, adaptive controllers are good at dealing with uncertain factors in unstructured environments. Usually, the adaptive controller consists of two loops for state feedback and parameter adjustment. The similarity between AIC and MRAC is that they both belong to the adaptive controller type.

### Experimental scene

This experiment simulates that the robotic arm will grab an object from one bookshelf and place it on another bookshelf. It mainly simulates actions instead of actually grabbing objects. The purpose of this is to observe as much as possible the performance of the controller without interference from other external objects. The last forward lean motion is to test whether the joints are in good working condition when the robot arm moves forward. In addition, the purpose of the robot arm moving to the left and right is to obtain the state of different joints when they move in the opposite direction, such as whether the position data is completely opposite, etc. The diversity of data provides strong evidence for controller performance analysis. During the experiment, all actions are repeated multiple times. This is because during the exercise test, sometimes the robot arm will have abnormal movements that are not easy to attract attention. To make the experimental data more convincing, every time during the test, motion will be executed multiple times, and then the observation data will be used for subsequent data analysis. These tasks are to obtain the position data and force data output by the robot arm during operation. The position data is used to analyze the accuracy and control efficiency of the controller to control the robot arm, for example, by analyzing time from one position to another. It can be seen that the controller controls the operating efficiency of the robotic arm. Effort data is mainly used to analyze the jitter of the robotic arm. It is noted that the consideration behind is that the essence of the robot arm is to change the position of an object, including its own joint position, therefore, such a simple test environment was designed, and this test result can be used in some similar environments, however, the environment changes greatly, for instance, in some very cluttered scenarios, more tests are needed to illustrate the results.

### Experiment on robustness

**Figure 1:**
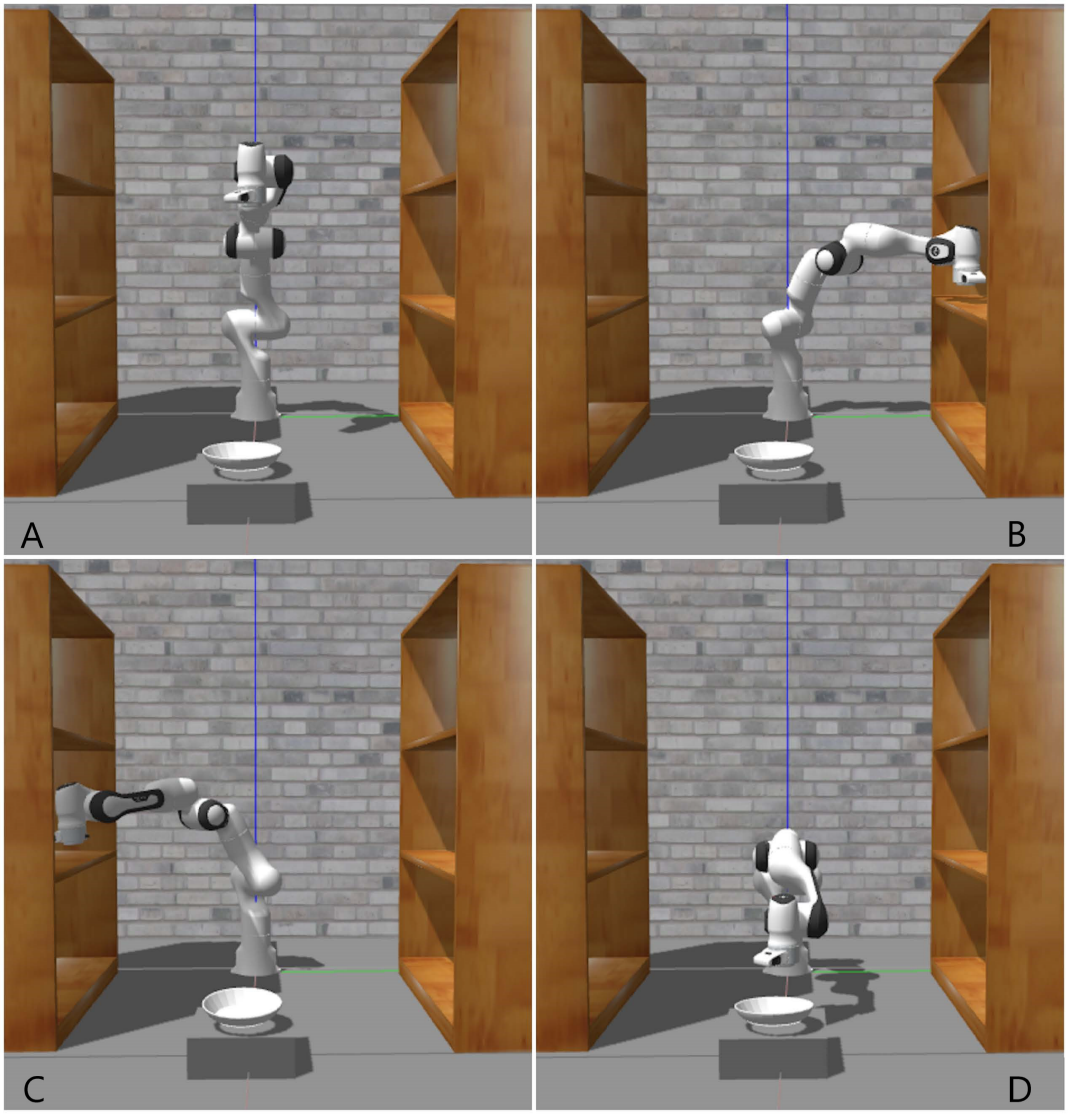
Simulation Scene: (A) Initial pose; (B) picking from the right shelf; (C) picking from the left shelf; (D) placing the object.

**Figure 2:**
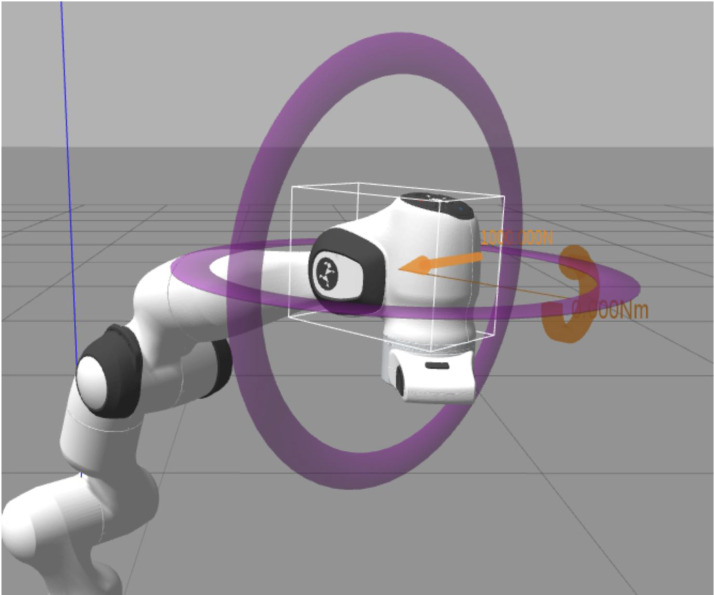
The direction and position of the external force on the robot arm.

The robustness test of the controller is by applying an instantaneous external force to a specific joint of the robot arm, because the robot arm will have a position shift or jitter after receiving external force. Then, by collecting joint position data, the time required for the robotic arm to return to a normal state or whether the controller can still control the robotic arm to work normally after receiving external force can be obtained. The joints that accept the external force are selected based on the changes in the position of the joints in space. The more obvious the changes in the position of the joints in space, the easier it is to see the changes in the data after being subjected to the external force. On the contrary, if you choose joint 1 or 2 which has no spatial position change, it is not easy to see the running status of the robot arm after force in the simulation. Finally, in the robustness test, there is another point that needs to be explained. The direction of the robot arm’s instantaneous force is opposite to the direction of the robot arm’s movement. Figures 1 and 2 are all the tasks of this experiment. The three action tasks in Fig. 1 are mainly to complete the general test of the basic controller and advanced controller. The purpose of the test in Fig. 2 is to complete the robustness of the controller. From Fig. 2, it can be seen that the robot arm is running in the right direction, but the external disturbance is left. The purpose of this design is to clearly see the robot arm’s response to external disturbance. In addition, if the direction of force is the same as the direction of movement of the robot arm, the adjustment of the robot arm’s controller to external disturbance may not be clearly observed unless a very large force is applied.

### PID controller adjustment

**Figure 3:**
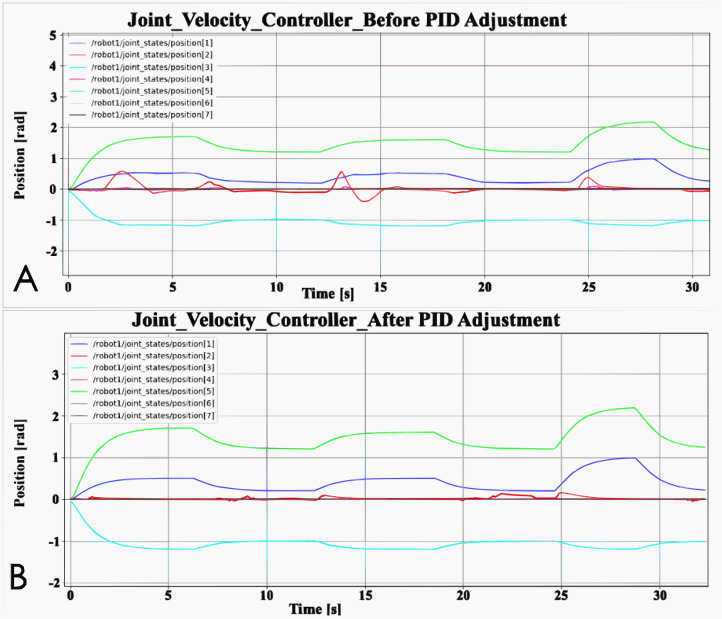
Comparison of PID controller before and after adjustment: (A) Joint velocity controller before PID adjustment; (B) Joint velocity controller after PID adjustment.

PID controller is composed of a proportional unit, integral unit and derivative unit. Adjust the performance of the PID controller by adjusting the three parameters. There are many ways to adjust PID, which can be adjusted manually or dynamically adjusted by machine learning. In this article, manual tuning parameters is used. The adjustment method is to adjust the proportional unit P first, then adjust the integral unit I, and finally adjust the differential unit D if necessary. In general, only adjust the parameters of P and I. In addition, the PID controller is just a simple controller with limited performance, so it cannot solve all control problems. In this article, there are two basic controllers that will be affected by PID parameters: joint Velocity Controller and Joint Position Controller. Taking the Joint Velocity Controller as an example, the comparison chart before and after PID parameter adjustment is shown in Fig. 3. Before the PID controller is adjusted, the robot arm cannot reach the set pose and there will be many poses that are not set. This means that the robot arm cannot be effectively controlled by the controller. In Fig. 3, before the PID controller is adjusted, the rotation angle of joint 2 often exceeds the given range, and its maximum value even exceeds that of joint 1. After adjusting the PID controller, the performance of the robot arm is obviously better. If the PID controller is not adjusted, the performance of the two basic controllers cannot meet the needs of the experiment, because the robot arm cannot reach the set posture during the simulation.

## Experiment Results

### Base controller benchmarking

**Figure 4:**
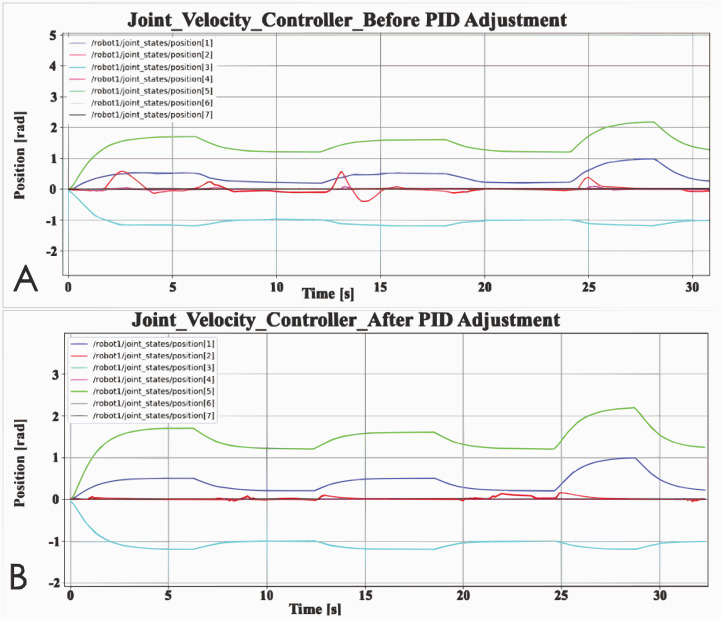
Basic controller position data: (A) Joint velocity controller before PID adjustment; (B) Joint velocity controller after PID adjustment.

**Figure 5:**
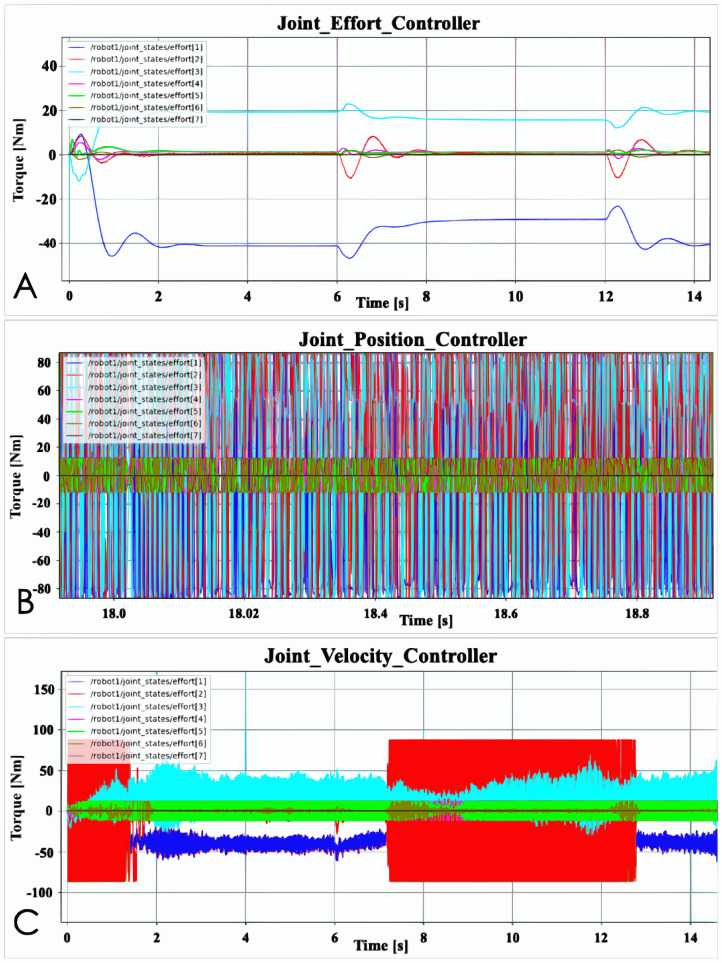
Basic controller effort data: (A) Joint effort controller; (B) Joint position controller; (C) Joint velocity controller.

Figure 4 shows the position data of the three basic controllers. It is clear to see from the figure that the data of the joints with the most obvious data changes are basically the same between different controllers. In the simulation process, these three controllers can also control the robot arm to reach the accurate position, and there is no too abnormal data, such as unable to reach the accurate position. But this result is after proper adjustment of PID parameters. It can also be seen from Fig. 4 that the robot arm takes the same time to complete the task designed in Fig. 1. In addition, the data output by the robot arm is the same whether it is at the inflexion point or at the maximum value. However, there are also joints with the significantly different performance data output, such as joint 2 and joint 4. Joint Position Controller has very obvious data anomalies between 0 and 3 s. Although Joint Velocity Controller also has this performance, it is far less obvious than Joint Position Controller. Furthermore, there are obvious data anomalies between 7–13 and 20–23 s, which caused the joints to shake at the target position during the simulation. However, the Joint Effort Controller does not have such abnormal data. Figure 5 shows the effort data of the three basic controllers.

It can be clearly seen in Fig. 5 that the Joint Effort Controller has no jitter data, and all the data is normal, but the other two controllers have obvious jitter data. During the simulation, the jitter of the robot arm controlled by the other two controllers can be seen from the simulation animation, but because the amplitude is not very large, it does not look obvious. However, the effort data clearly shows the jitter. Although both the Joint Position Controller and the Joint Velocity Controller are jittering, the Joint Velocity Controller does not make some joints shake significantly at certain times. For example, there is no serious jitter problem for joint 2 between 2 and 7 s. The jitter problem of Joint Position Controller basically lasts for the entire task process, and all joints have jitter. Since displaying the status of 7 joints at the same time is too confusing for the data of the Joint Velocity Controller and Joint Position Controller, then joint 4 is selected for specific data display. Since the position data shows that the degree of change of joint 4 is not very large, the jitter value will be relatively small. If a joint with a very large value is selected, it will be difficult to read the jitter data.

**Figure 6:**
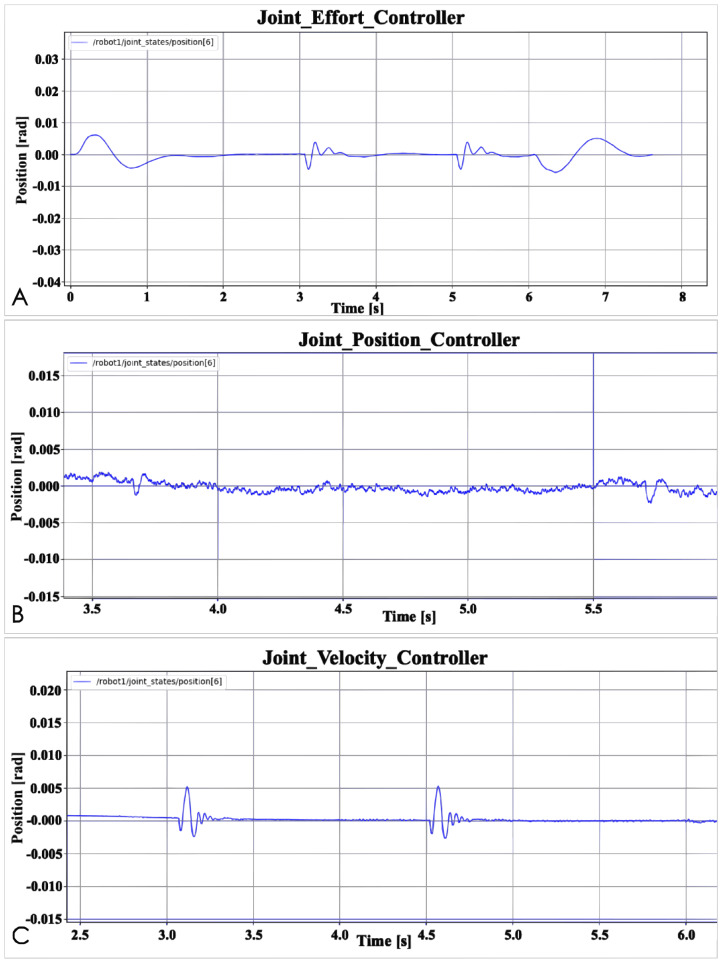
Basic controller robustness test: (A) Joint effort controller; (B) Joint position controller; (C) Joint velocity controller.

The last test is a robustness test. Figure 6 shows the result. The purpose of this test is to observe the time it takes for different controllers to respond to external interference before recovering the interference, and whether the robot arm can return to a normal state. In order to be more intuitive, Table 1 records the response time of the three basic controllers and the time of accept external force. “Accept external force” means the time when the robot arm is subjected to an external force in the experiment. In the experiment, the robot arm is accepted to two times external forces. “Response time” means how long it takes for the controller to restore the robot arm to its previous state when the position of the joint changes. According to the final result, the Joint Position Controller takes the shortest time, and the Joint Effort Controller takes the longest time. Of course, this does not mean that the Joint Position Controller is better than the Joint Effort Controller. The specific reasons will be explained in the data analysis section. In addition, even if the time is different, the difference is only 0.1 s, so the robustness test of the three controllers is equivalent to obtaining relatively good results. First, the three controllers restore the robot arm to the normal state and the time it takes is all short, secondly, these three basic controllers have the ability to restore the robot arm to normal state. The accuracy of the robotic arm can be determined by observing whether each joint of the robotic arm reaches the correct position. By observing the force of each joint, it can be seen whether the robot arm is running stably.

**Table 1:** Robustness testing of basic controllers.

Controller	Time	
	Accept external force (s)	Response time (s)
Effort controller	3 and 5	0.3
Position controller	3.7 and 5.7	0.1
Velocity controller	3.1 and 4.5	0.2

### Advanced controller benchmarking

**Figure 7:**
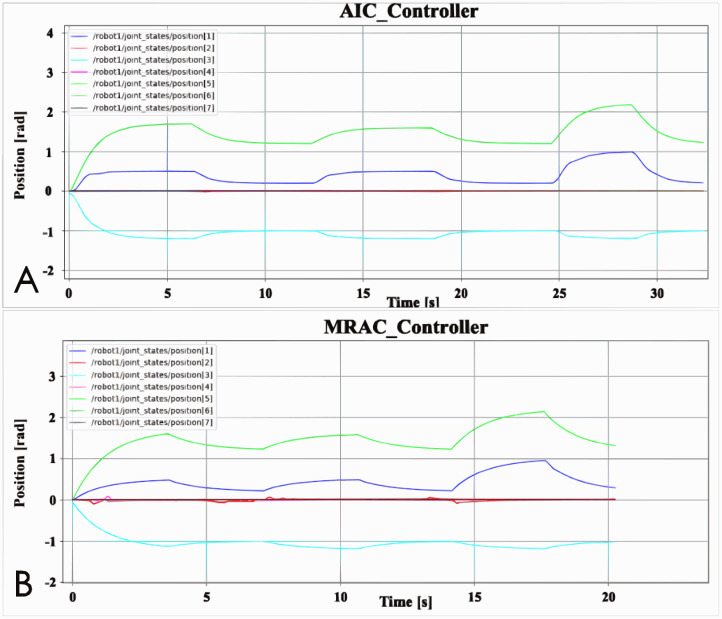
Advanced controller position data: (A) AIC controller; (B) MRAC controller.

Figure 7 shows the position data of the experimental results of the advanced controller benchmarking of this article. Through this data, the accuracy and control efficiency of the advanced controller AIC and MRAC to control the robot arm is obtained. Figure 7 also shows that it takes less time for the MRAC to control the robot arm to reach the designated position than the AIC to control the robot arm to reach the designated position. The AIC controller takes 30 s but the MRAC only takes 20 s. Furthermore, the turning point of each line in the figure is the position data of the joint when the robot arm reaches the specified position, so whether it is in the simulation animation or the joint output data can prove that the two advanced controllers can control the robot arm to the specified position because the value of the turning point is the same. However, the joint 2 data in Fig. 7 has obvious differences. The fluctuation of joint 2 of the robot arm controlled by MRAC is more obvious than that of AIC. For the convenience of observation, the data of joint 2 is taken out separately. Since the data of joint 4 in Fig. 7 is not obvious, the data of joint 4 is also taken out.

**Figure 8:**
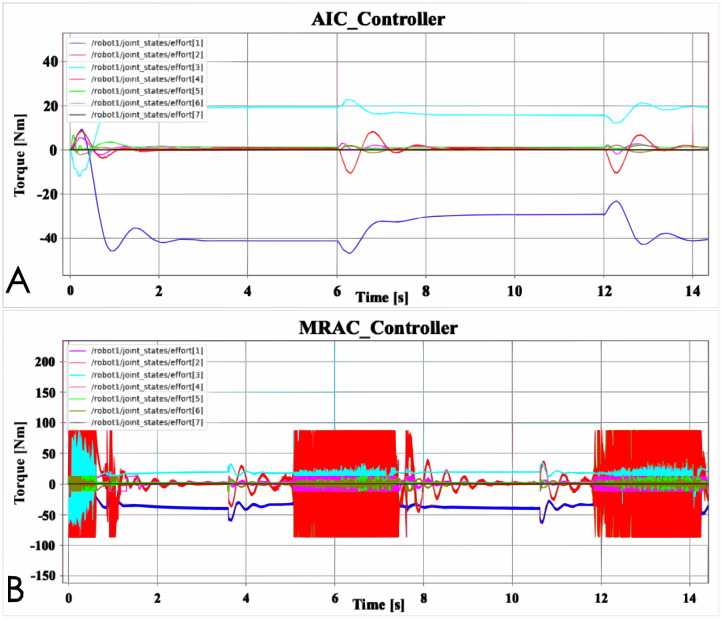
Advanced controller Effort data: (A) AIC controller; (B) MRAC controller.

Figure 8 is the Effort data figure of AIC and MRAC. It can be seen from the figure that the jitter of the robotic arm controlled by the MRAC controller is much more serious than the AIC, which is not obvious in the position data. Furthermore, when the AIC controlled robotic arm has data fluctuations, the MRAC controlled robotic arm also has data fluctuations, such as at 6 and 12 s. The difference is that MRAC is sometimes earlier than AIC. This may be because the robot arm controlled by MRAC runs faster. For MRAC, the problem of joint 2 is more serious, and the jitter of joint 2 in the simulation animation is indeed more obvious. Although other joints also jitter, the gap is still relatively large compared with joint 2. Compared with MRAC, there is almost no jitter in the robot arm controlled by AIC, and the data change law of AIC is smoother, and the data change of MRAC is more chaotic.

**Figure 9:**
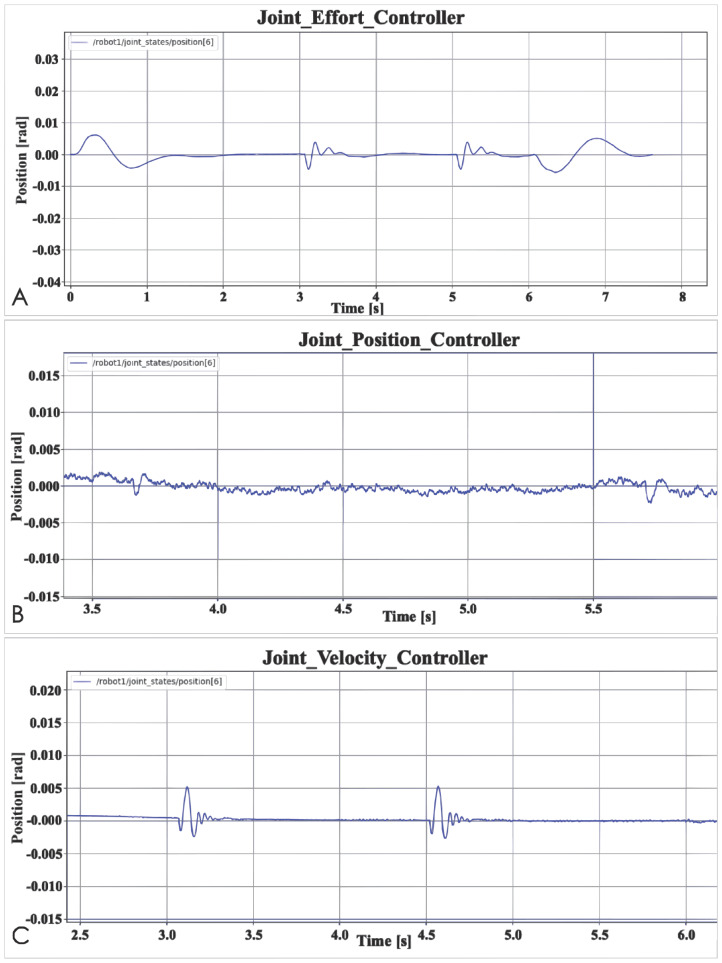
Advanced controller robustness test: (A) Joint effort controller; (B) Joint position controller; (C) Joint velocity controller.

Figure 9 shows the robustness test results of the advanced controller AIC and MRAC. The direction and position of the robot arm bearing the external force have been shown in Fig. 2. Furthermore, the movement direction of the robot arm is to the right, and the robot arm will receive an external force to the left. Furthermore, it is not difficult to see from Fig. 9 that AIC reacts more smoothly to external interference, while MRAC is more chaotic. This phenomenon should be because the jitter of MRAC is more obvious than that of AIC, which leads to more chaotic MRAC data output. Another phenomenon is worth noting. The time interval between two data fluctuations of the robot arm controlled by AIC is about 5 s, for example, between 1 and 6 s. The time interval of MRAC is 2 s, for example, 5–7 s. This phenomenon should be caused because the robot arm controlled by AIC runs slower than the robot arm controlled by MRAC. In order to better display the experimental results, relevant experimental data are recorded in Table 2.

**Table 2:** Robustness testing of advanced controllers.

Controller	Time	
	Accept external force (s)	Response time (s)
AIC controller	3 and 5	0.3
MRAC controller	6.4	0.1

The final result shows that the robot arm controlled by the AIC controller needs 0.3 s to recover to the previous state after receiving the external force, while the MRAC only needs 0.1 s. In addition, the output data value of the MRAC after the external force is smaller than the value of AIC a lot of. However, this does not mean that the robustness of MRAC is better than that of AIC controller. This experimental result can only prove that the two advanced controllers can adjust the robot arm to the state before the external force is applied. The data size and response time output by the robotic arm may also be affected by other factors, such as jitter. More analysis will be explained in the data analysis section.

## Discussion

### Based controller

According to the experimental results of the basic controller, several conclusions can be drawn:

- The basic controller can control the robot arm to the designated position, and the time spent is the same;
- The basic controller has jitter, but the jitter of the Joint Effort Controller is the least obvious, and the other two basic controllers have obvious jitter;
- All three basic controllers can restore the robot arm interfered by an external force to the state before the interference, but the response time of the controller is different.

Based on the previous introduction to the basic controller, it is not difficult to see that the two controllers that perform poorly (with obvious jitter) are affected by the PID parameters, so the adjustment of the PID parameters will directly affect the performance of the basic controller. It is challenging to obtain very good results based on the adjustment of PID parameters because there are seven joints that need to adjust PID parameters in this robot arm, and the joints will affect each other during the adjustment process. For example, when the PID parameters of joint 1 are adjusted to a satisfactory condition, the PID parameters of joint 2 are adjusted, but during the adjustment process, it is found that the data output of joint 1 has changed again. Therefore, in this experiment, adjust the PID parameters as much as possible. The result is that each joint can work, and every PID parameter adjustment is not forced to obtain very good results. So PID parameters affect the performance of the two basic controllers. In addition, the poor performance of the controller may also due to the controller itself. The Joint Velocity Controller and Joint Position Controller accept speed and position values as input data, and then output as Effort commands through PID mapping. First, that also proved the conclusion of the experiment, that is, PID parameters affect the performance of the basic controller, and besides, because the data needs to be mapped and converted, this may also cause problems with the controller, as the data conversion may cause errors. The better-performing Joint Effort Controller does not need to consider these issues. Because the input and output of the Joint Effort Controller are both Efforts data, it does not need to map input and output data, nor is it affected by PID parameters, so this may be a reason of the better performance of the Joint Effort Controller. Although the performance of the two basic controllers is not very good, it is only reflected in the occurrence of jitter. More importantly, the three basic controllers can spend the same time controlling the robot arm to the designated position.

This test also did a robustness test on the controller, the purpose is to observe whether the controller is capable of restoring the controller interfered by external forces to the state before the interference. From the results, the three controllers all meet the requirements. The difference is that the response time and output value of the controller are different. This should be caused by the jitter problem of the robot arm itself. It can be seen from the Effort data output by the robotic arm that there is jitter between the joints. When the robot arm is subjected to external force, the force generated by the jitter and the jitter direction will affect the magnitude of the external force. As a result, the controller’s response and time to external force interference in different results. Although the result data is different, this robustness test proves that the basic controller has the ability to deal with simple external disturbances and restore the robot arm to the state before the disturbance.

### Advanced controller

Active inference controller and MRAC are the advanced controllers selected for this controller benchmarking. From the results, both controllers can control the robot arm to reach the preset posture, but they take different time. MRAC is obviously faster to complete the task than AIC. According to the characteristics of the AIC controller, some parameters need to be adjusted adaptively within the AIC. Most of the parameters of MRAC are preset, so this may be one of the reasons that cause the MRAC controller to execute faster than AIC. In addition, the jitter problem of MRAC controller is obviously more obvious than that of the AIC controller. According to the understanding of MRAC, this may also be due to the parameter setting of MRAC itself. In Fig. 1, it can be seen that the data output by the plant will be input to the controller, which is MRAC, which compares the data with the reference model internally. This process will produce errors, which will be passed to the adaptive algorithm module, and then the recalculated result is still with error, and finally, the result of the controller output is with error, this result is passed to Actuator and Plant. Since the model parameters are set in advance, any inappropriate situation occurs, the internal parameters of the controller will not be changed. Therefore, if the MRAC jitter problem is solved, the MRAC parameter setting and the reference model setting should be very accurate. But in general, the results of this benchmark test can show that these two controllers can meet the basic requirements of controlling the operation of the robotic arm. Although the time is different, the two advanced controllers can control the robotic arm to reach the specified pose.

This benchmarking also designed a simple robustness test for the advanced controller. According to the results, it can be seen that the AIC response to the external force is smoother, and it took more time to restore the robot arm to the state before the external force interference. Like the basic controller, although MRAC can control the robotic arm to return to the state before the interference in a short time, due to the jitter problem of MRAC itself, it does not mean that MRAC is more robust than AIC. If the further testing is needed, the jitter problem should be solved first, whether it is a basic controller or an advanced controller, this is necessary, the purpose of this is to minimize the impact of other uncertainties on the experiment.

## Future Works

There are still some works that can be improved in our future work. First, improving PID parameter adjustment, because this will not only improve the performance of the controller but also help the test to obtain more accurate data. The adjustment can choose learning-based method because this can achieve the purpose of dynamic adjustment, especially beneficial for the seven interactive PID parameter adjustments. The purpose of PID parameter adjustment is to reduce the jitter problem, so it is also possible to choose to introduce other controllers to solve the jitter problem. In addition, adding grippers to the robotic arm and adding objects with actual weight makes the environment more complicated in order to obtain more experimental data. Objects can be unfixed or stationary or add multiple robots to complete tasks in coordination to test the performance of the controller. More tasks and benchmarking methods can be developed in the future.

## Conclusions

The purpose of this article is to perform benchmark tests on a 7-DOF robotic arm with various controllers. According to the results, the tests have achieved the requirements. First, choose the simulated pick and place task to complete the test of the controller’s control robot arm accuracy, control efficiency and jitter problems. The selection of controllers also covers two types of controllers: advanced controllers and basic controllers, and appropriate analysis is carried out based on the test results and the controller itself. In addition, during the experiment, all tasks were repeated many times, and the data was presented in the article as the result of this experiment. In summary, this experiment completed the benchmarking of the two types of controllers, both the completion of the preset experiment and the data collection have been successfully demonstrated.

## Additional Information and Declarations

### Data Availability

The following information was supplied regarding data availability: Data is available at GitHub: https://github.com/evanevanevan/Robot-control-benchmarking. Code is also available at GitHub: https://github.com/cpezzato/panda_simulation.
